# Masticatory efficiency, bite force and electrical activity of the masseter and temporalis muscles in bodybuilders

**DOI:** 10.4317/jced.58368

**Published:** 2021-09-01

**Authors:** Murilo-César-Bento-Laurindo Júnior, Marcelo-Coelho Goiato, Fernanda-Pereira de Caxias, Karina-Helga-Leal Turcio, Emily-Vivianne-Freitas da Silva, Michel-da Silva Deusdete, Daniela-Micheline dos Santos

**Affiliations:** 1 DDS, MSC. Department of Dental Materials and Prosthodontics, Araçatuba School of Dentistry, São Paulo State University-UNESP. Road Jose Bonifacio 1193, Vila Mendonca, Aracatuba. São Paulo, Brazil; 2 DDS, MSC, PhD. Department of Dental Materials and Prosthodontics, Araçatuba School of Dentistry, São Paulo State University-UNESP. Road Jose Bonifacio 1193, Vila Mendonca, Aracatuba. São Paulo, Brazil; 3 BPE. KM Runner Incentivo Esporte, Araçatuba, São Paulo, Brazil

## Abstract

**Background:**

This study aimed to compare the masticatory efficiency, the maximum voluntary occlusal bite force (MVOBF) and the electrical activity (EMG) of masticatory muscles of practitioners of upper limb bodybuilding before and after physical activity.

**Material and Methods:**

Twenty healthy individuals (10 men and 10 women, age from 18 to 30 (mean of 24.7 years old) without masticatory system disorders, that regularly practice hypertrophic physical activity were submitted to the analyses of masticatory efficiency, MVOBF, and surface EMG of the temporalis and masseter muscles. The masticatory efficiency was analyzed by comminution of the artificial material (Optocal®) and a sieving method. The MVOBF was measured by a dynamometer, and EMG was evaluated during resting mandibular position, maximum voluntary clenching (MVC), and MVC with a Parafilm M tape between teeth, and free mastication of chewing gum. The analyses were made before (T0) and immediately after the performance of upper limb bodybuilding exercises (T1). The data of masticatory efficiency and MVOBF were submitted to the Student T-test, and their correlations were analyzed by the Pearson correlation test, and the EMG data were submitted to the 2-way repeated measures ANOVA, all tests with a 5% significance.

**Results:**

There was a significant decrease of masticatory efficiency after the training. No statistical difference in the MVOBF and EMG was found, and there was a positive correlation between masticatory efficiency and MVOBF.

**Conclusions:**

Therefore, changes were found for masticatory efficiency only, which decreased after hypertrophic exercise. There was a positive correlation between masticatory efficiency and MVOBF.

** Key words:**Masticatory efficiency, bite force, physical activity, electromyography.

## Introduction

The population is ever more interested in improving quality of life, aesthetics, and general health. For a satisfactory quality of life, there is a group of contributing factors, such as the regular practice of physical activity (1). Ample access to information related to corporal aesthetics leads many individuals to physical activity, for health and muscle growth, either on their own or in weight-training institutions. Hypertrophy training are exercises that require great physical force, so that there is an increase of muscular force and volume (2).

The stomatognathic system consists of a group of structures that forms a complex system to perform swallowing, phonation, and mastication (3). Authors have related the stomatognathic system with certain sports, such as cycling, boxing, martial arts, rugby, diving, and athletes in general, principally associating these sports with dental clenching and consequent temporomandibular dysfunctions (TMD) (4-6). However, according to the knowledge of the authors, strength training and hypertrophy still have not been related to the stomatognathic system functioning, including the analyses of masticatory efficiency, maximum bite force, or electrical activity of masticatory muscles.

Masticatory efficiency consists of breaking down of food into smaller pieces by mastication and it can reflect masticatory function (7). There are different methods for measuring masticatory efficiency, such as partitioning in sieves through the use of natural foods or silicone-based synthetics (Optocal®), synthetic foods in which the glucose is the measurable particle, and the colorimetric method (8,9). Considered a gold-standard method, partitioning in sieves has been widely used (10), and Optocal® has been the material of choice (11). Bite force is related to quality of life, since it is able to influence the nutritional quality of individuals, relating itself to masticatory efficiency, milling of food and its digestion (12). Therefore, the evaluation of muscle force during mastication is an important complement to the masticatory efficiency analysis (13).

Training related to resistance and muscular force requires maximum activity that leads to muscle fatigue, which can be analyzed by means of surface electromyography (EMG), which associates an increase in amplitude of the electronic signals to fatigue (14). This analysis is widely used to evaluate the efficiency of rehabilitation treatments and the TMD related to myofunctional alterations (15), allowing the study of characteristics during oral function execution. Changes were observed in the electric activity of the masticatory muscles in sport climbers compared to non-climbers, which suggests that periodontal mechanoreceptors and electrical activities can be interrelated (16).

This study aimed to compare the masticatory efficiency, maximum voluntary bite force (MVOBF) and EMG of masticatory muscles of practitioners of upper limb bodybuilding before and after physical activity to evaluate changes in these functions immediately after physical exercises. The hypotheses were that there would be a decrease in masticatory efficiency and MVOBF after practice; there would be a positive correlation between masticatory efficiency and MVOBF; and there would be alterations in EMG of muscles after practice.

## Material and Methods

-Participants and study design

The Human Research Ethics Committee approved this study. Participants signed the Free Informed Consent form and experiments were conducted according to the ethics principles.

Young university students that frequented weight-training institutions in Aracatuba, Sao Paulo, Brazil, and that performed hypertrophic training were invited to participate in the study.

Participants selected were examined at the Department of Dental Materials and Prosthodontics and the inclusion criteria were: good general health or mild systemic illness, as defined by the American Society of Anesthesiologists (17); good cognitive ability; practice hypertrophic physical activity at least three days a week, being at least 1 hour each training, for at least one year; be fully dentate; not use psychotherapeutic treatment, muscular relaxants, or androgenic anabolic steroids; not possess any type of degenerative chronic illness; not possess a history of facial trauma or muscular lesions; not use any type of dental prosthesis or orthodontic apparatus; and be at least 18 years old, with a maximum age of 30, as well as not present any symptom of TMD confirmed by the Research Diagnostic Criteria (RDC/TMD) (18) by which the collection of demographic data was performed.

All participants were submitted to the analyses of masticatory efficiency, MVOBF, and surface EMG of temporalis and masseter muscles, before (T0) and immediately after the performance of a physical training exercises (T1).

-Masticatory efficiency

The masticatory efficiency was evaluated by means of mastication of artificial food based on the fabrication of Optocal® (10,11). Components were mixed and placed in acrylic molds with cylindrical perforations (12 mm in diameter by 5 mm of height), to later be stored in an incubator at 65°C for 16 hours, guaranteeing complete polymerization. Each participant received 3 g of artificial food that was masticated for 35 cycles, monitored by the examiner. This quantity of cycles is very close to the swallowing moment (13). After mastication, milled particles were expelled from the oral cavity into a group of 3 sieves. Then, the participant rinsed the oral cavity to eliminate remaining particles, expelling them in the same sieves. Finally, an intra-oral inspection was performed to certify the no remnant of food remained in the oral cavity.

Particles contained in the sieves were washed with water and dried in an incubator at 50°C for an hour. Then, sieves were placed in a vibrator for 60 seconds, and particles were separated according to the wire meshes of the sieves, with openings of 1,70 mm / 1,18 mm / 0,42 mm, coupled in descending order of the opening and held by a metallic base. The content of each sieve was weighed separately (10,13) to five decimal places using a digital analytical balance (Ohaus, SP, Brazil). Finally, after weighing, the value (in grams) taken from the 3 sieves was subtracted from 3 g to obtain the quantity of Optocal® that passed through the sieves. The larger the value of the “leftovers” of the artificial food, that is, the larger the quantity that passed through the 3 sieves, the larger was the capacity of milling the food, and thus, greater masticatory efficiency.

-MVOBF

The MVOBF was measured by the IDDK dynamometer (Kratos – Equipamentos Industriais Ltda, Brazil) with 15 mm of thickness and 1000 N. The examiner received thorough training to be familiar with the apparatus, and participants were also familiarized. Recordings were performed in the posterior region (first molar area) of each side (12). Each recording was done three times for each region, with the participants instructed to bite the transducer with maximum strength. Each record was made for approximately 5 ± 2 seconds, with an interval of 2 minutes (12). Recordings were collected, and the most elevated MVOBF recordings was selected.

-EMG

The Trigno Wireless Foundation System electromyograph (ADInstruments, Australia), was used to capture and transmit the recordings to the LabChart software. Trigno Mini EMG surface electrodes were used for the recording and attached with Trigno Mini Sensor Adhesive (ADIntruments, Australia). Regions that would receive the electrodes were washed with water and soap. Next, a light rubbing was performed with gauze embedded with 70% alcohol for the removal of skin oiliness (19). A ground electrode was positioned on the wrist (19). In the temporalis, electrodes were attached to the fiber long axis, perpendicularly, and distances of 1.5 to 2 cm from the inferior margin of the zygomatic arch. In the masseter, electrodes were positioned in the muscle central point, equidistant from their areas of attachment. The positions of the electrodes in the first session were marked in the medical records of each participant and registered in a “template” of transparent acetate for ink-jet A4 without a stripe (Filiperson Ind. de Papéis Ltda., Rio de Janeiro, Brazil), for the relocation of electrodes in the same positions in different sessions.

The signal recording was done in the following order: Initial rest of mandibular position for 10 seconds (R1); Maximum voluntary clenching (MVC) with parafilm M tape (Bemis Flexible Packaging, Neenah, WI, USA) for 10 seconds (P); MVC without parafilm for 10 seconds (MVC1); The participants chewed sugarless gum (mint flavor, Trident®, Mondelez, Bauru, Brazil), free and uninterrupted, for 10 minutes; Rest for 10 seconds after mastication (R2); MVC without parafilm for 10 seconds (MVC2); The participants performed a 10-minute break; Rest for 10 seconds after the break (R3); MVC without parafilm for 10 (MVC3).

-Physical Training

After the initial tests, participants performed a training that was drafted and applied by a graduated and licensed physical educator. Specific exercises had the same series of equipment for all of individuals, in a standardized manner for men and women, aiming for muscular hypertrophy; however, in different intensities, respecting the limits of each participant, but requiring great physical force.

Seeking greater reliability of results and a greater control of activities, the physical educator accompanied all training. Exercises were performed in 3 series of 8 to 12 repetitions close to exhaustion, with a pause of 60 seconds between the series, and 120 seconds from one exercise to the next. The equipment and activities done were: Bench press with barbell, Abdominal row, Inclined dumbbell bench press, Back press, Lumbar hyperextension, Neutral position articulated rowing, Barbell biceps curl, Triceps pulley, Standing lateral dumbbell press.

The physical educator was instructed to observe signs of masseter muscle contraction and the facial expressions of the participants. Immediately after the physical training, analyses were repeated.

-Statistical Analysis

The sample size estimation was made based on a pilot study, to guarantee the reliability of the study. The GPower 3.1 software (Heinrich-Heine-Universität Düsseldorf, Germany) was used, which indicated that for the MVOBF analysis, the N necessary would be 5 participants (β = 0.2% and α = 0.05%).

The statistical analysis was performed using the SPSS 22.0 program (Chicago, USA). The masticatory efficiency and MVOBF values were submitted to the Student T-test, and the Pearson correlation was applied to verify the correlation between them (*p* = 0.05). For the MVOBF analysis, the mean was calculated from the values obtained for the right and left sides. The EMG data were submitted to the 2-way repeated measures variance ANOVA (*p* = 0.05). The ANOVA tests were performed separately for each muscle (temporalis and masseter) and analysis (rest and MVC), considering the factors: Period (T0 and T1) and analysis (R1, R2 and R3 or MVC1, MVC2 and MVC3).

## Results

Twenty participants (10 men and 10 women), with ages of 18 to 30 (mean: 24.7 years) were included. No drop-out happened and no data was missed.

**Figure 1:**
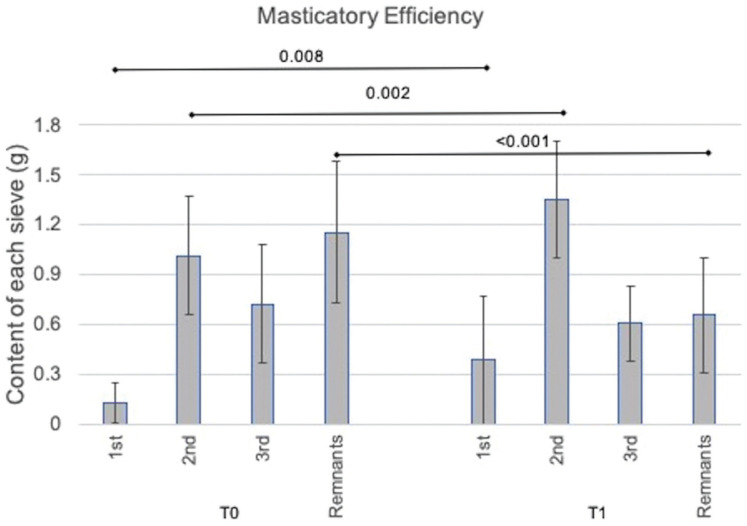
Graphic representation of data of masticatory efficiency. Content of each sieve and remnants (mean and standard deviation) in grams at T0 and T1.

**Figure 2:**
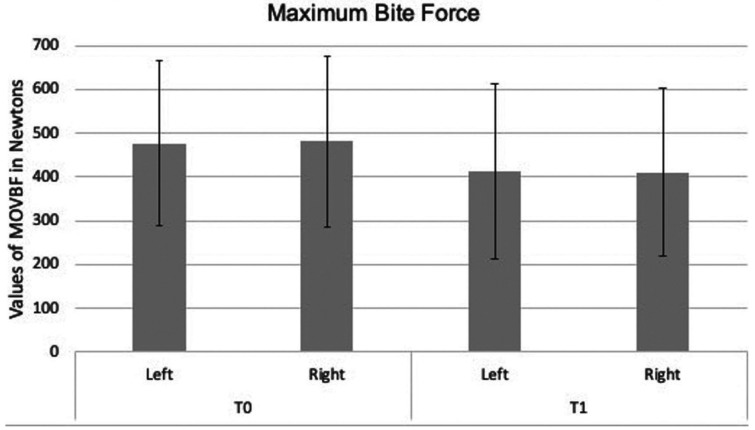
Data of MVOBF (N) at T0 and T1 on both sides.

Representation of data obtained for the analysis of masticatory efficiency and MVOBF, before (T0) and after training (T1), are found in Figures 1 and 2.

For the analysis of masticatory efficiency, a difference was encountered between the periods of analysis, with a decrease of weight in the “leftovers” in the post-training period (*p* < 0.001) (Table 1, Fig. 1). In addition, a significant increase in weight can be observed in the post-training period for the 1st (*p* = 0.008) and 2nd (*p* = 0.002) sieves (Fig. 1). Regarding MVOBF, no significant difference was found for MVOBF between T0 and T1 (Table 1, Fig. 2).

**Table 1:**
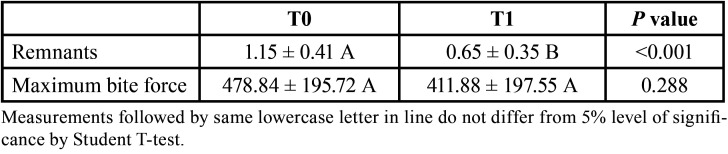
Mean values ± standard deviation of food remnants (g) and maximum bite force (N) before (T0) and after training (T1).

A positive correlation was observed between masticatory efficiency and MVOBF; that is, the greater the MVOBF, the greater the “leftovers” and the masticatory efficiency (Table 2).

**Table 2:**
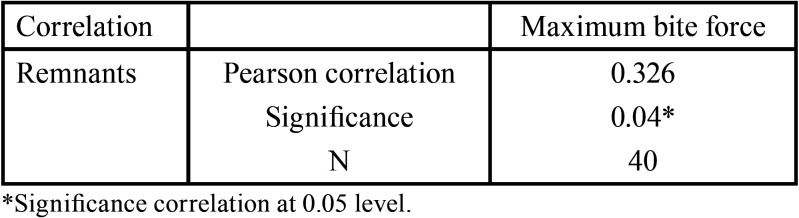
Correlation between masticatory efficiency and maximum bite force of individuals evaluated.

The electromyographic data were normalized by the activity of the respective muscles in MVC with parafilm (Table 3). For temporalis muscle, there was no statistical difference between T0 and T1 for rest (*p* = 0.857) nor for MVC (*p* = 0.861). For masseter muscle, there was no statistical difference between T0 and T1 for rest (*p* = 0.328) nor for MVC (*p* = 0.773).

**Table 3:**
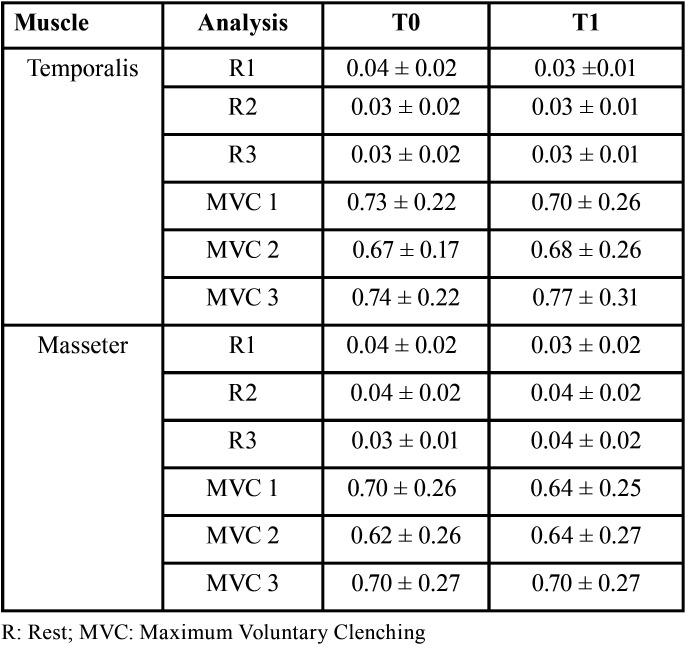
Mean values ± standard deviation of temporalis and masseter muscular activity (mV) at rest before (T0) and after training (T1) in different analyses.

## Discussion

The hypothesis that there would be a decrease in the masticatory efficiency and the MVOBF after practice of hypertrophic activity was partially rejected since there was a significant decrease of only the masticatory efficiency; the hypothesis that there would be a correlation between both the analyses was accepted; and there would be alterations in the electrical activity of the temporalis and masseter muscles after practice of hypertrophic activity was rejected. The hypertrophic training demanded effort and required high muscular contraction (2) which could lead to fatigue of trained muscles, which is characterized by a decrease in muscular activity when the level of oxygen saturation decreases to a point where anaerobic metabolism begins (20). Athletes that practice strength sports seem to present changes in bioelectric potentials of masticatory, in addition, the role of these muscles and their periodontal mechanoreceptors is important to the athletes’ performance (21). By requiring intense effort during training, athletes could have contractions of masticatory muscles, since typical facial expressions, such as a frowned forehead or face, can be observed during high physical effort, leading to an increase of muscular activity (4). Besides that, the increase of activity of mastication muscles could also be caused by dental clenching, which is common during an intense exercise. However, the present study did not aim to assess the presence of oral habits during physical activity because there are already other studies which showed that strength exercises, mainly in upper limb, cause contraction of masticatory muscles during exercises (16), and that the role of periodontal mechanoreceptors and the contraction of masticatory muscles in force production and muscle activity is somehow relevant in the context of athletic performance (16).

This study had a decrease in masticatory efficiency after training, and despite no occurrence of a significant reduction in MVOBF, there was a positive correlation between the reduction of efficiency and MVOBF. Ginszt *et al*. 2020 (16) verified higher bioelectrical activities of the masseter muscles during maximum voluntary clenching in sport climbers when compared to controls, and suggest that functional activity within the masseter muscles appears to be associated with the climbing. Based on their results, the climbers’ masseter appears to exert greater activity during tightening or function. This could explain why the bodybuilders of the present study did not show a reduction in the bite strength after exercising, since they probably have muscle characteristics similar to the climbers. Therefore, the physical activity performed did not change the functional capacity of the volunteers’ muscles. In the present study, myoelectric activity did not decrease after exercises, and the reduction in masticatory efficiency cannot be explained by electrical activity, and other factors need to be explored and studied. The act of mastication can be considered complex, since it involves the coordinated action of the tongue, mandible, and masticatory muscles, besides stimulating the secretion of saliva (22). Its force is important for the development of facial bones and depends on the periodontal ligament proprioception, and its efficiency is important for the health of individuals, since when there are masticatory difficulties, a tendency of reduced consumption of fibers and the risk of nutritional problems and gastrointestinal illnesses occur (23). The decrease of masticatory efficiency found demonstrates the decrease in milling capacity of the artificial food, without the decrease in myoelectric activity. It is important to note that the milling of food depends on factors such as the tooth shape, characteristics of food breakage, and the coordination of mandible muscular activity responsible for generating bite force (24). In the present study, the decrease in the reduction in crushing capacity cannot be attributed to muscle electrical activity. Thus, other factors should be studied, such as salivary composition and formation of by-products after intense physical exercise, which could influence on the chewing ability.

 The bite force can be influenced by different factors such as the presence or non- presence of muscular pains, and/or inflammation in the temporomandibular articulation, facial osteoporosis, gender, number of teeth, and education level, which is related to the standard of oral health (25,26). It is the same physiological characteristic that is related to the quality of life. The analysis of the present study demonstrates that one session of high intensity exercise was not sufficient to alter the MVOBF, but caused its numerical decrease. It is worth highlighting that the absence of statistical difference does not necessarily imply clinical irrelevance; a factor in which the clinical relevance of the alteration of bite force and its positive correlation with masticatory relevance can be based. It is also important to report that the masticatory muscles could have presented smaller masticatory capacity due to an energy deficiency after the intense training, since important changes in the intracellular concentration of a number of metabolites occur during fatiguing activities. The change in metabolism and the strength reduction correlate due to some factors, such as the decrease of high energy compounds that lead to the reduction of energy dependent processes and the accumulation of degradation products with harmful effects (24). In the present study, the masticatory and MVOBF tests were performed immediately after the training. Participants did not have the opportunity to replace the metabolites, by feeding themselves.

The presence of positive correlation between the masticatory efficiency and MVOBF observed is the target of controversies, since other studies have reported that the relation between the masticatory efficiency and the MVOBF is minimal (9) while other studies have shown an intimate and direct relationship (26). Some authors affirm that an adequate bite force is one indicator of efficient masticatory function (25). They affirm that masticatory efficiency involves 4 principal characteristics which are: applicated bite force; contact between adjacent teeth; mandibular movements; and mastication duration. Such codependency deserves attention, since mandibular movements and mastication duration were not evaluated.

The decrease of masticatory efficiency after training could suggest a possible muscle fatigue and is an important factor for high performance athletes, since their post-training nourishment should be of easy and rapid absorption for the muscle capacity recuperation, including masticatory muscles. Thus, if the masticatory capacity is smaller after intense trainings, these athletes should consider this fact, including the form of food ingestion, if solid or liquid, since the form could influence their thermogenesis, depending on physical activity (27).

The authors are unaware of any other study which evaluates not only the behavior of the head and neck muscles, but also the masticatory efficiency and bite force in individuals who practice hypertrophic physical activity. Based on this, it is important to perform studies evaluating both aspects for a more profound comprehension of the masticatory system behavior in these individuals. The present study provided information about the effects of this specific type of physical training on the masticatory system, which could help orientate athletes, trainers, and nutritionists, about the possible decrease in masticatory capacity and the most adequate manner of post-training alimentation. Thus, the individual will better perform the activity and obtain more satisfactory results and avoid overloading the stomatognathic system.

## Conclusions

A decrease of masticatory efficiency was observed after hypertrophic exercise in upper limb bodybuilders, but neither the MVOBF nor electrical activity of the masticatory muscles underwent significant changes. There was a positive correlation between masticatory efficiency and MVOBF.
